# Muscle Thickness and Function of Transversus Abdominis and Gluteus Medius in Individuals with Chronic Non-Specific Low Back Pain

**DOI:** 10.3390/jcm15020666

**Published:** 2026-01-14

**Authors:** Thanawat Yodthee, Patraporn Sitilertpisan, Aatit Paungmali, Sompong Sriburee, Samatchai Chamnongkich, Amornthep Jankaew, Ranida Quiggins, Cheng-Feng Lin

**Affiliations:** 1 Integrated Neuro-Musculoskeletal, Chronic Disease, and Aging Research Engagement Center (ICARE Center), Department of Physical Therapy, Faculty of Associated Medical Sciences, Chiang Mai University, Chiang Mai 50200, Thailand; tanawat_yo@cmu.ac.th (T.Y.); aatit.p@cmu.ac.th (A.P.); samatchai.c@cmu.ac.th (S.C.); amornthep.jan@cmu.ac.th (A.J.); 2 Department of Radiology Technology, Faculty of Associated Medical Sciences, Chiang Mai University, Chiang Mai 50200, Thailand; sompong.sriburee@cmu.ac.th; 3 Department of Anatomy, Faculty of Medicine, Chiang Mai University, Chiang Mai 50200, Thailand; ranida.quiggins@cmu.ac.th; 4 Department of Physical Therapy, College of Medicine, National Cheng Kung University, Tainan 70101, Taiwan; connie@mail.ncku.edu.tw

**Keywords:** low back pain, transversus abdominis, gluteus medius, ultrasound imaging, motor control

## Abstract

**Background**: Non-specific low back pain (NSLBP) is associated with altered neuromuscular control of the lumbopelvic–hip complex (LPHC). However, the functional behavior of the transversus abdominis (TrA) and gluteus medius (GM) during upright postural tasks, with and without the abdominal drawing-in maneuver (ADIM), remains unclear. This study aimed to compare TrA and GM activation between individuals with NSLBP and asymptomatic controls during standing and single-leg stance using rehabilitation ultrasound imaging (RUSI). **Methods**: Thirty-two participants (16 with NSLBP and 16 asymptomatic controls) underwent RUSI assessment under four task conditions: standing and single-leg stance, with and without ADIM. Muscle function was quantified using thickness change derived from ultrasound measurements. A two-way mixed-model analysis of variance with Bonferroni-adjusted post hoc comparisons was performed. **Results**: Significant group × condition interactions were identified for TrA activation (*p* < 0.05). Individuals with NSLBP demonstrated reduced TrA activation during standing with ADIM and reduced GM activation during single-leg stance compared with asymptomatic controls. The effect sizes were moderate to large for TrA activation and small to moderate for GM activation. **Conclusions**: These findings suggest task-specific differences in neuromuscular activation patterns in individuals with NSLBP. Ultrasound-derived thickness change measures obtained during functional, weight-bearing tasks may provide clinically relevant information to support motor control rehabilitation strategies.

## 1. Introduction

Low back pain (LBP) remains one of the most prevalent musculoskeletal disorders globally and is a leading contributor to disability and socioeconomic burden [1]. Lifetime prevalence of LBP exceeds 70%, with substantial prevalence observed across adult age groups [2]. Non-specific low back pain (NSLBP), the most common subtype [3], refers to back pain without a specific identifiable pathology [4]. Despite the complex nature of NSLBP, postural instability and chronic symptoms have been frequently related to impaired neuromuscular control of the deep abdominal and hip stabilizing muscles [5,6].

The transversus abdominis (TrA) and gluteus medius (GM) are key stabilizers contributing to lumbopelvic control during upright and unilateral tasks [7,8]. The TrA modulates anticipatory stiffness and assists in controlling trunk orientation when postural demand increases, whereas the GM contributes to frontal-plane pelvic control, especially during unilateral loading tasks. Altered activation of these muscles may influence trunk-pelvic alignment and force transfer [9]. Reduced TrA activation has been reported in individuals with NSLBP [10], while altered GM function has been associated with impaired lateral pelvic control during asymmetrical loading tasks such as single-leg stance [11,12].

RUSI enables real-time visualization of muscle morphology [13]. It is widely applied to assess muscle thickness in non-weight bearing conditions, especially supine and side-lying positions [14,15], but its application during specific functional tasks remains unclear. TrA activation typically decreases during upright tasks compared with non-weight-bearing positions [14], whereas GM activation depends more directly on task-specific mechanical demands. Thickness change reflects mechanical muscle behavior associated with contraction rather than direct neural activation [16].

The abdominal drawing-in maneuver (ADIM) is widely used in clinical practice to enhance TrA activation and recruit other deep stabilizers within the LPHC. Previous studies have demonstrated that ADIM can improve activation of both TrA and GM in asymptomatic and LBP individuals [17,18]. However, its effectiveness during functional tasks such as standing and single-leg stance remains unclear. Although previous studies examined TrA or GM activation separately, no research has systematically examined simultaneous TrA and GM activation during upright functional tasks with and without ADIM in individuals with NSLBP. This gap limits understanding of task-specific neuromuscular control during functional, weight-bearing postural demands in individuals with NSLBP.

This study aimed to compare TrA and GM activation between individuals with NSLBP and asymptomatic controls during standing and single-leg stance, with and without ADIM. We hypothesized that individuals with NSLBP would demonstrate task-specific differences in muscle activation under higher postural demands. Understanding how these muscles respond to combined voluntary activation and postural demands may help clarify task-specific neuromuscular deficits and support the development of motor control-based rehabilitation programs.

## 2. Materials and Methods

### 2.1. Study Design

**Figure 1:**
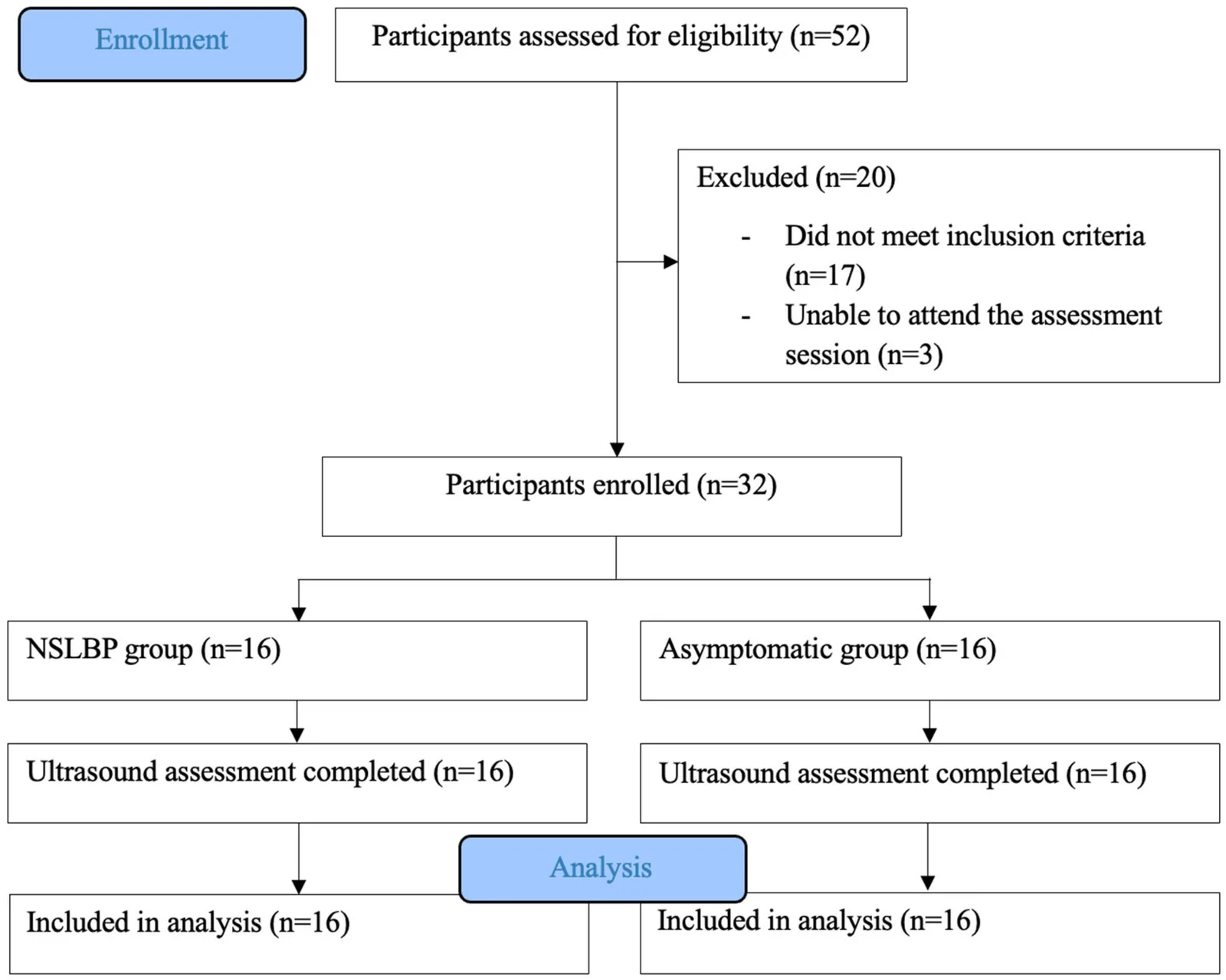
Study procedure.

This research utilized a cross-sectional study design to understand TrA and GM muscle morphology and function in functional tasks (standing and single-leg standing) in individuals with NSLBP compared with asymptomatic individuals. The study was conducted between July and September 2025 following the STROBE guideline (Figure 1).

### 2.2. Sample Size Justification

Sample size was calculated using G*Power 3.1 based on partial η^2^ = 0.26 [15], targeting 80% power and α = 0.05 for detecting interaction effects. A minimum of 32 participants was required.

### 2.3. Participants

A total of 32 participants, 16 with NSLBP and 16 asymptomatic, were recruited. Male and female participants were eligible if they were aged 20–59 years old with a BMI between 18.5 and 24.9 kg/m^2^ and met all inclusion criteria. All participants were recruited from physical therapy clinics, communities, and social media. Following approval from the institutional human research ethics review board and obtaining individual informed consent, participants were requested to complete the demographic data questionnaire.

### 2.4. Asymptomatic Group

Participants had no history of LBP within the previous year. Exclusion criteria included a history of hip, knee, or back pain that interfered with activities of daily living (e.g., getting in and out of bed, getting up and down stairs, using the restroom on their own, or picking up objects from the ground) or any history of hip or lumbosacral spine surgery, major injuries, lower extremity injury or surgery within the last 12 months, and high physical activity level and exercise ≥ 3 times a week.

### 2.5. NSLBP Group

Participants had pain between the 12th rib and buttocks with and without referred pain to one or both legs [19]. NSLBP is either unilateral or bilateral for at least 3 months. The average pain intensity is ≥3/10 and ≤8/10 on the Visual Analog Scale (VAS) in the past week [20]. The Oswestry Disability Index is >20 out of 100 [21]. Exclusion criteria include any prior surgery to the lumbosacral spine, pregnancy, spinal pathology (such as spinal tumor, fracture, infection, and osteoporosis), neurogenic signs on clinical examination [22], inability to stand or perform single-leg stance, or the presence of neurological signs or clinical red flags [23], having participated in any specific training of the core stabilization or hip muscles in the last 6 months, and high physical activity level and exercise ≥ 3 times a week [24].

### 2.6. Abdominal Drawing-In Maneuver Practice

Participants practiced ADIM using a standardized protocol emphasizing deep abdominal engagement without thoracic compensation or excessive pressure fluctuation. A pressure biofeedback unit (PBU) (Stabilizer Pressure Biofeedback-Chattanooga Group, Hixson, TN, USA) was used. The PBU was placed between the second lumbar and first sacral spine levels. The pressure gauge was set at 40 mmHg, and participants were instructed to maintain a pressure reduction of approximately 4–10 mmHg during ADIM.

### 2.7. RUSI Measurement

A real-time ultrasound scanner (Canon Xario-100S, TUSX100S model, Otawara, Tochigi, Japan) equipped with an 8 MHz transducer was used in B-mode. Depth was standardized at 4.0 cm for TrA and 6.0 cm for GM. Gain was fixed at 52% (kept constant for all participants) with the focal zone positioned at the interface of muscle layers. Measurements were collected while the participant was in a standing position for TrA and GM measurements in resting and single-leg standing, with and without ADIM. The transducer was placed on the symptomatic side in the NSLBP group and on the dominant side in asymptomatic controls. Minimal probe pressure was maintained to avoid tissue deformation. A curvilinear probe was used in B-mode, and three images were captured for each condition. All ultrasound images were coded and randomized. The order of ADIM and non-ADIM trials was randomized to minimize order effects. The assessor measuring thickness was blinded to participant group. RUSI measurements were performed by a licensed physical therapist. Prior to the study, the examiner completed a minimum of 10 h of hands-on training under the supervision of a musculoskeletal radiologist and a physical therapist with 5 years of experience in RUSI. This training was followed by practice on a minimum of 20 volunteers.

### 2.8. Transversus Abdominis Ultrasonographic

**Figure 2:**
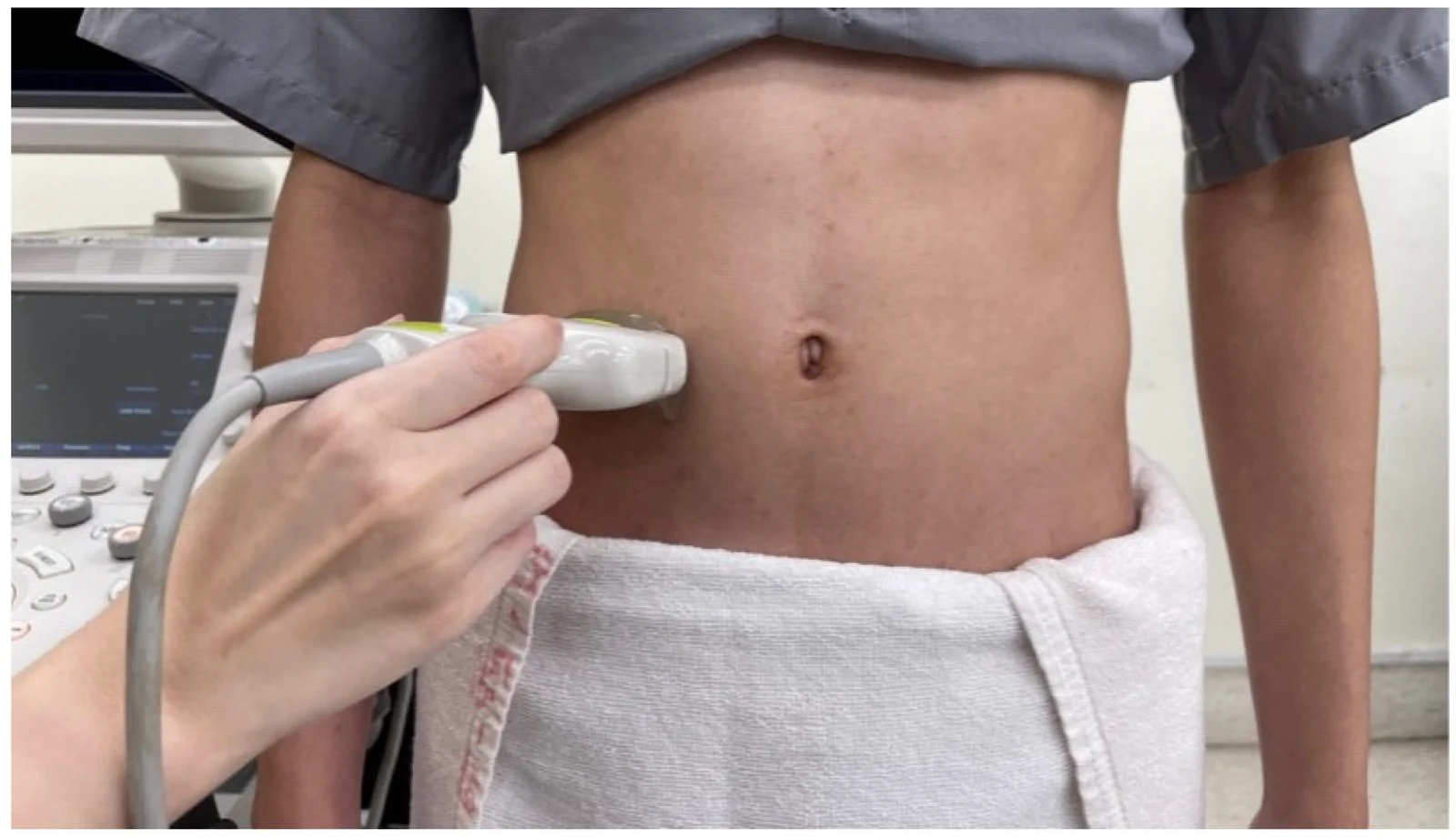
Location of TrA ultrasonographic.

Markers were positioned 10 cm lateral to the umbilicus (Figure 2). The TrA ultrasound was captured at the end of expiration. For single-leg standing, participants raised the asymptomatic or non-dominant leg to 90° of knee flexion while abducting their arms to 45° and extending their elbows to maintain balance. Each measurement was held for 5–6 s with a 40–50 s rest between trials. Resting measurements were taken first, followed by randomized trials of single-leg standing with or without ADIM, with 30–60 s breaks to prevent fatigue. Standardized ADIM instructions were provided to ensure proper technique without rib elevation, pelvic tilting, or breath holding during the procedure.

### 2.9. Gluteus Medius Ultrasonographic

**Figure 3:**
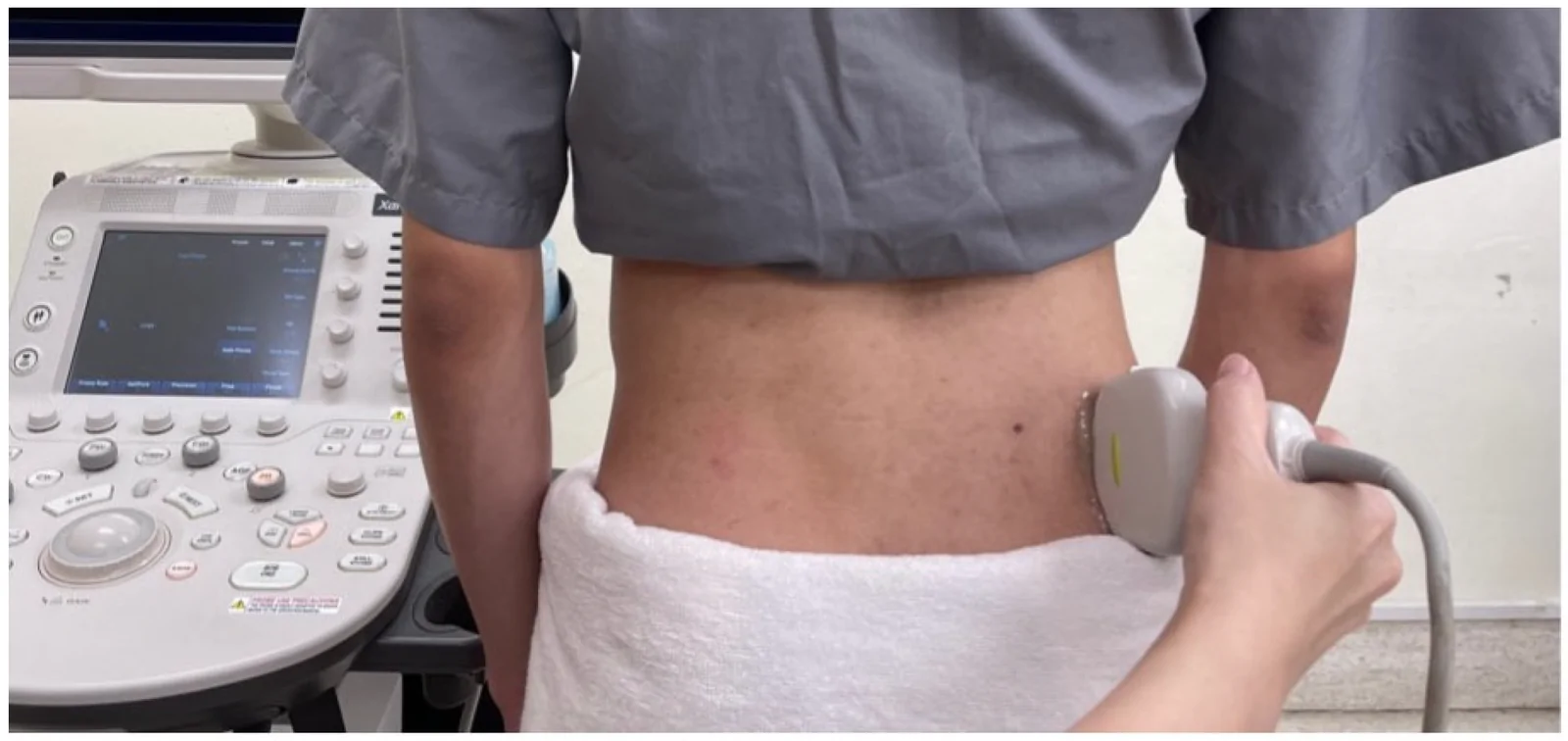
Location of GM ultrasonographic.

The transducer was placed on the lateral hip, positioned along the lower half of a coronal line extending between the ASIS and PSIS to the greater trochanter’s apex (Figure 3). Ultrasound images were captured at rest and during single-leg standing with and without ADIM, following procedures similar to those used for TrA evaluation.

### 2.10. Muscle Thickness and Function Measurement

**Figure 4:**
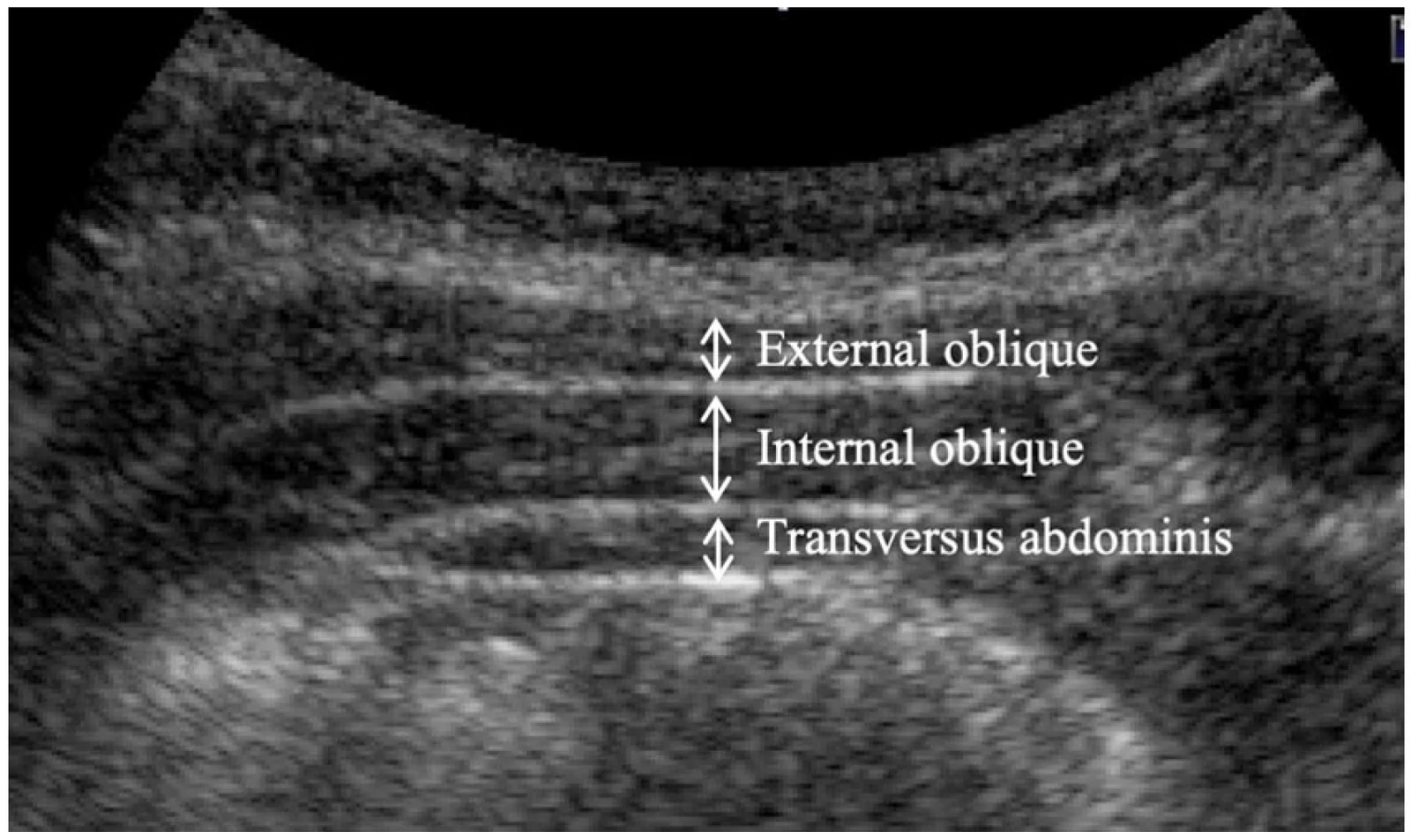
TrA muscle thickness measurement.

**Figure 5:**
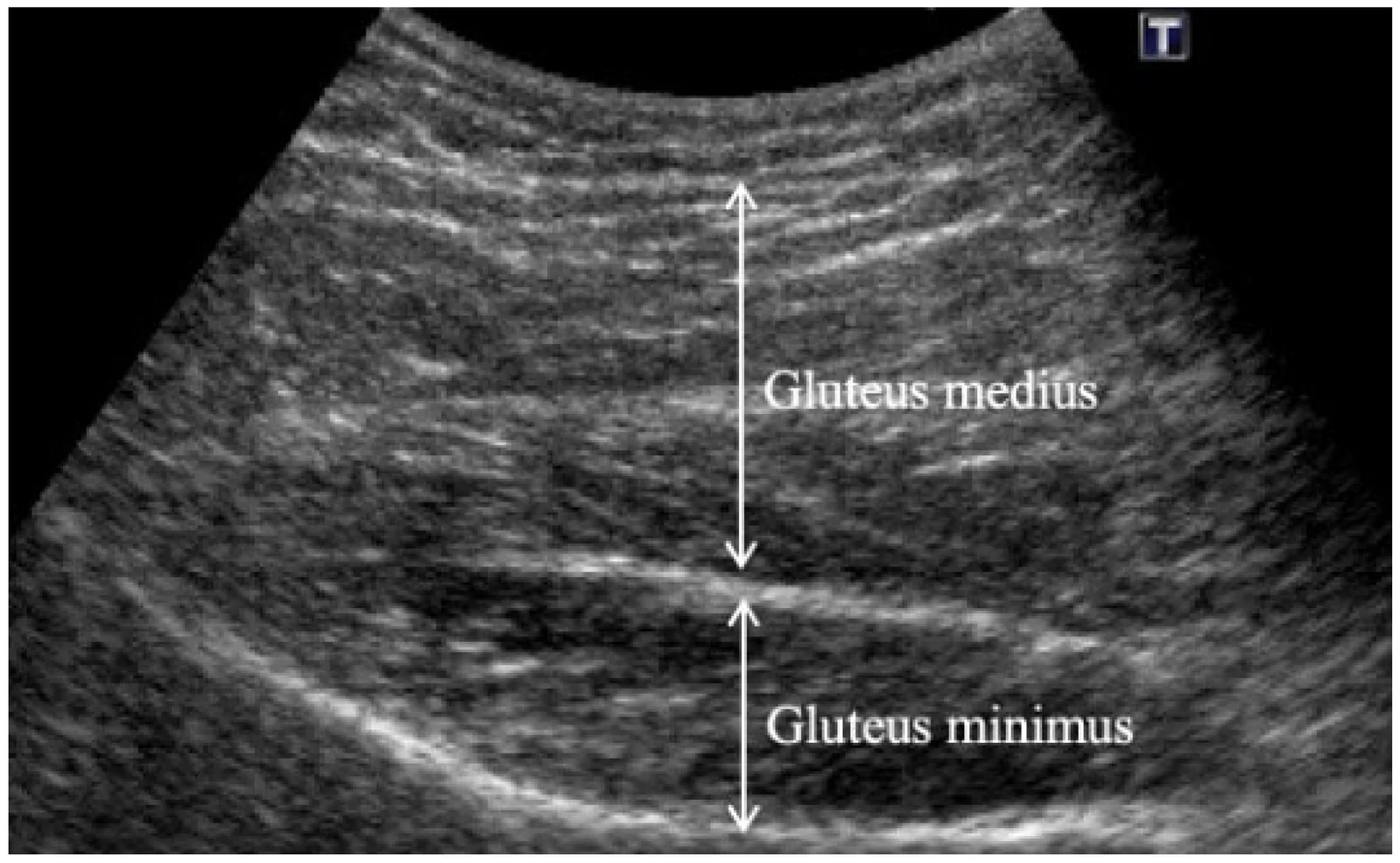
GM muscle thickness measurement.

All images were saved and quantified offline using the MicroDicom software (standard version 2025.3) [25] for measuring muscle thickness (Figure 4 and Figure 5). To avoid distortion, all measurements were taken in the center of the picture and reported in millimeters. The average of three images was used for each condition [19]. The muscle function (muscle thickness change) formula was used to determine the difference in thickness between muscle resting and contraction: (thickness contracted − thickness rest)/(thickness rest) [16].

### 2.11. Data Analysis

Assumptions of normality were tested using the Shapiro–Wilk test. Descriptive statistics were used to describe participant demographic data and outcomes. Outliers were screened using boxplot inspection, and no values were removed. The analysis was performed based on the study design as a two-way mixed-model ANOVA. Homogeneity of variance was examined using Levene’s test. Post hoc pairwise comparisons were corrected using the Bonferroni method to control Type I error. The magnitude of all significant effects was quantified using partial eta squared (η^2^), and all mean differences were reported with 95% confidence intervals (95% CI). Effect sizes (Cohen’s d) were calculated for all pairwise post hoc comparisons to quantify the magnitude of differences. Effect sizes were interpreted as small (0.2), medium (0.5), and large (0.8). Sphericity was assessed using Mauchly’s test and Greenhouse–Geisser correction was applied when violated. Normality was also verified through visual inspection of Q-Q plots. All analyses were performed using IBM SPSS Statistics (version 30) (IBM Corp., Chicago, IL, USA), with the significance level set at <0.05.

## 3. Results

### 3.1. Participant Demographic Characteristics

The demographic and baseline characteristics, including age, sex, and body mass index (BMI), showed no significant differences (*p* > 0.05) between the asymptomatic control group and the NSLBP group (Table 1).

### 3.2. Comparison of Main Effect Estimates for TrA and GM

Descriptive and inferential results are summarized in Table 2, Table 3, Table 4 and Table 5. Table 2 summarizes the main and interaction effects for TrA and GM muscle thickness across conditions and groups.

### 3.3. Mean Difference (95% CI) in Muscles Thickness Between Groups

The mean differences between the NSLBP and asymptomatic groups for each condition, derived from Bonferroni post hoc comparisons, are presented in Table 3. Standing with ADIM demonstrated the largest between group difference (*p* = 0.005) for TrA.

**Table 3:** Mean difference in TrA and GM thickness (mm). The mean difference values were derived from post hoc Bonferroni comparisons. CI = 95% confidence interval.

Muscles	Conditions							
	Standing Without ADIM	*p*-Value	Standing with ADIM	*p*-Value	Single-Leg Without ADIM	*p*-Value	Single-Leg with ADIM	*p*-Value
TrA	0.26 (−1.085, 1.60)	0.511	1.67 (0.55, 2.79)	0.005	0.08 (−0.64, 0.80)	0.810	0.80 (0.12, 1.48)	0.023
GM	−0.83 (−4.40, 2.74)	0.772	−1.32 (−4.90, 2.26)	0.510	−1.46 (−5.12, 2.20)	0.460	−0.33 (−3.90, 3.25)	0.660

TrA = transversus abdominis, GM = gluteus medius, ADIM = abdominal drawing-in maneuver.

### 3.4. The Thickness Change in TrA and GM in Standing and Single-Leg Standing with and Without ADIM

The muscle function, quantified as the thickness change in TrA and GM, is presented in Table 4. GM thickness change was significantly reduced in the NSLBP group during single-leg standing (*p* = 0.007).

**Table 4:** The thickness change (functional activation) of TrA and GM.

Muscles	Conditions	NSLBP	Asymptomatic	Mean Difference with 95% CI	*p*-Value
TrA	Standing	0.23 ± 0.28	0.69 ± 0.64	−0.46 (−0.82, −0.10)	0.014
	Single-leg standing	0.19 ± 0.44	0.33 ± 0.32	−0.14 (−0.42, 0.14)	0.328
GM	Standing	0.001 ± 0.09	−0.01 ± 0.09	0.01 (−0.06, 0.08)	0.744
	Single-leg standing	−0.05 ± 0.05	0.02 ± 0.08	−0.07 (−0.12, −0.02)	0.007

NSLBP = non-specific low back pain group, Asymptomatic = asymptomatic control group, TrA = transversus abdominis, GM = gluteus medius.

### 3.5. ADIM-Related and Posture-Related Activation Effects for TrA and GM Thickness

ADIM-related and posture-related activation effects were calculated to quantify task-specific thickness changes in the TrA and GM (Table 5).

**Table 5:** TrA and GM muscle thickness for ADIM-related and posture-related effects.

Muscles	Effect Type	Conditions	NSLBP	Asymptomatic	*p*-Value
TrA	ADIM-related	Standing	0.55 ± 0.91	1.96 ± 1.77	0.008
		Single-leg	1.96 ± 1.77	1.13 ± 1.48	0.106
	Posture-related	Without ADIM	0.30 ± 0.89	0.13 ± 0.77	0.567
		With ADIM	0.17 ± 0.88	−0.69 ± 1.43	0.046
GM	ADIM-related	Standing	0.04 ± 2.99	−0.43 ± 2.32	0.614
		Single-leg	−1.78 ± 1.71	0.34 ± 2.21	0.005
	Posture-related	Without ADIM	2.08 ± 3.45	1.45 ± 2.95	0.584
		With ADIM	0.25 ± 3.45	2.23 ± 2.53	0.074

NSLBP = non-specific low back pain group, Asymptomatic = asymptomatic control group, TrA = transversus abdominis, GM = gluteus medius, ADIM = abdominal drawing-in maneuver.

## 4. Discussion

This study investigated the thickness and thickness change in the TrA and GM muscles in individuals with NSLBP and asymptomatic controls. Furthermore, the effects of ADIM and postural demand on muscle activation were calculated to estimate differences in task-specific muscle responses between individuals with NSLBP and asymptomatic controls. The key findings of this study were that individuals with NSLBP demonstrated task-specific differences in TrA activation, particularly during standing with ADIM, and reduced GM activation during single-leg stance compared with asymptomatic controls.

A significant interaction between group and condition suggested that differences in TrA activation were more evident during voluntary control. Under higher postural demand, activation may rely more on reflexive mechanisms, reducing group differences [20,21]. The TrA appears to be especially sensitive to pain-related disruptions of motor control, even though both deep and global stabilizers respond to postural load. While the NSLBP group demonstrated a limited capacity to adapt activation when postural demands increased, asymptomatic participants demonstrated greater adaptation of TrA activity across conditions.

Thickness change analysis demonstrated task-specific differences in neuromuscular responses. Individuals with NSLBP showed reduced TrA activation during upright stabilization and reduced GM activation during single-leg stance, which may reflect impaired coordination between local and global stabilizers. These findings suggest that neuromuscular responses in NSLBP may be task-dependent and may reflect difficulty integrating voluntary contraction with increasing postural demand rather than generalized muscle weakness.

Previous electromyographic studies have demonstrated that trunk muscle activation is influenced by changes in task demands and loading conditions in individuals with and without low back pain [22]. Studies examining different exercise and movement tasks have shown that increasing postural or mechanical load, such as during limb lifting or unilateral lower extremity tasks, is associated with increased activation of core musculature as measured by electromyography. In particular, exercises involving leg lifting have been reported to enhance activation of trunk stabilizing muscles and improve core muscle function, suggesting task-dependent modulation of motor control strategies [23]. These electromyographic findings are consistent with the present results, which demonstrated task-specific differences in muscle thickness during conditions with increased postural demand. However, direct comparisons should be interpreted with caution due to differences in measurement modalities, as electromyography assesses neural activation whereas ultrasound-derived thickness change reflects mechanical muscle behavior.

Clinically, these results suggest that motor control retraining may be considered as a complementary approach alongside strengthening interventions [24]. Early interventions may consider incorporating ADIM during upright tasks to facilitate TrA engagement.

These findings suggest that ADIM may be considered as one component of task-specific motor control strategies rather than as an isolated intervention [25]. However, interventional studies may further clarify whether improving TrA and GM activation enhances functional outcomes. In addition, thickness change measures were more sensitive than thickness in detecting deficits in muscle activation [15] and are suggested as a useful outcome in clinical practice.

A strength of this study is the focus on functional, weight-bearing postures. This study evaluated muscle activation in standing and single-leg positions, which better reflect postural control during functional activity. Understanding LPHC function requires concurrent consideration of both TrA and GM activation. Reliability in standing and single-leg was established through studies using reliability protocols, which ensured high consistency. However, as RUSI cannot capture neural activity, it remains an indirect assessment for muscle activation. Ultrasound-based muscle thickness assessment during functional tasks reflects task-related changes in muscle behavior. As no additional assessment method, such as electromyography, was included, the present findings describe muscle behavior within the context of the tasks performed in this study. Although physical activity level was controlled through the inclusion and exclusion criteria, psychosocial factors such as fear avoidance and pain catastrophizing were not assessed and may have influenced motor control strategies. Although the observed differences in muscle thickness were modest, the findings provide task-specific insight into muscle behavior during functional, weight-bearing postures.

In summary, both ADIM and postural load significantly affect TrA and GM activation, though in different ways. Individuals with NSLBP demonstrated lower TrA activation during ADIM and reduced GM activation during single-leg stance compared to asymptomatic controls, suggesting impaired coordination of the LPHC. Asymptomatic individuals showed greater muscle responsiveness and more consistent control across postures, indicating more efficient neuromuscular regulation. Functional retraining that integrates ADIM with hip abductor activation may enhance muscle control. This combined approach may also improve spinal stability.

Evaluating TrA and GM thickness under postural conditions can provide clinically useful information about motor control impairments in NSLBP. Muscle thickness and thickness change metrics can support clinical decision-making and facilitate monitoring of rehabilitation progress. Clinically, these findings suggest that TrA activation should be assessed in upright ADIM tasks and GM activation should be evaluated during single-leg stance to identify task-specific deficits. This study evaluated simultaneous TrA and GM activation under combined ADIM and postural demands, providing novel insight into task-specific motor control impairment in NSLBP.

## 5. Limitations

This study has some limitations. The results cannot be generalized as clinical outcomes and since this was a cross-sectional analysis, causal relationships between pain and neuromuscular deficits cannot be inferred. RUSI provides only an indirect estimate of muscle activation and lacks resolution of neuromuscular control. Future studies using combined ultrasound and electromyography might quantify muscle activation directly. The intervention effect of LPHC muscle activation with longitudinal or interventional designs is needed to clarify the activation effect and therapeutic impact in both standing and single-leg standing positions.

## 6. Conclusions

ADIM and postural loading significantly affected TrA and GM activation. Individuals with NSLBP showed reduced and less coordinated thickness change responses, especially in TrA during standing with ADIM and in GM during single-leg stance. These task-specific alterations may reflect altered lumbopelvic motor control strategies, and RUSI-derived thickness change measures may assist clinicians in identifying activation deficits during functional assessment.

## Figures and Tables

**Table 1:** Participant demographics and baseline clinical characteristics.

Characteristics	Asymptomatic (Mean ± SD)	NSLBP (Mean ± SD)
Age, years	32.00 ± 11.28	30.56 ± 8.06
Sex	M: 8 (50%), F: 8 (50%)	M: 8 (50%), F: 8 (50%)
BMI	22.19 ± 2.87	21.84 ± 1.64
Leg dominance (right side n, %)	12 (75%)	11 (68.75%)
Side of low back pain (n, %)		
Unilateral	-	10 (62.5%)
Bilateral	-	6 (37.5%)
ODI Score	-	30.77 ± 7.35
Low back pain intensity (VAS Scale)	-	6.52 ± 0.84

M = male, F = female, BMI = body mass index, ODI = Oswestry Disability Index, VAS = Visual Analog Scale, Asymptomatic = asymptomatic control group, NSLBP = non-specific low back pain group.

**Table 2:** Main and interaction effects for TrA and GM muscle thickness across task conditions.

Muscle	Condition	Group		Main Effect				Interaction Effect (Group × Condition)	η^2^_p_
		NSLBP	Asymptomatic	Group	η^2^_p_	Condition	η^2^_p_		
**TrA**	Standing without ADIM	2.97 ± 0.75	3.22 ± 2.13	F (1, 30) = 1.13, *p* = 0.296	0.296	F (3, 90) = 15.78, *p* < 0.001	0.345	F (3, 90) = 5.43, *p* = 0.002	0.153
	Standing with ADIM	3.52 ± 0.82	5.19 ± 3.42						
	Single-leg without ADIM	3.27 ± 0.90	3.36 ± 1.85						
	Single-leg with ADIM	3.70 ± 0.84	4.49 ± 3.12						
GM	Standing without ADIM	31.10 ± 5.92	30.26 ± 8.60	F (1, 30) = 0.09, *p* = 0.772	0.003	F (3, 90) = 6.21, *p* = 0.002	0.171	F (3, 90) = 1.73, *p* = 0.181	0.055
	Standing with ADIM	31.14 ± 6.85	29.83 ± 8.27						
	Single-leg without ADIM	33.18 ± 6.71	31.72 ± 7.75						
	Single-leg with ADIM	31.39 ± 6.57	32.06 ± 7.63						

η^2^_p_ = partial eta square, NSLBP = non-specific low back pain group, Asymptomatic = asymptomatic control group, TrA = transversus abdominis, GM = gluteus medius, ADIM = abdominal drawing-in maneuver.

## Data Availability

The data presented in this study are available from the corresponding author upon reasonable request.
