# Correction: Yodthee et al. Muscle Thickness and Function of Transversus Abdominis and Gluteus Medius in Individuals with Chronic Non-Specific Low Back Pain. *J. Clin. Med.* 2026, *15*, 666

**DOI:** 10.3390/jcm15207855

**Published:** 2026-10-12

**Authors:** Thanawat Yodthee, Patraporn Sitilertpisan, Aatit Paungmali, Sompong Sriburee, Samatchai Chamnongkich, Amornthep Jankaew, Ranida Quiggins, Cheng-Feng Lin

**Affiliations:** 1 Integrated Neuro-Musculoskeletal, Chronic Disease, and Aging Research Engagement Center (ICARE Center), Department of Physical Therapy, Faculty of Associated Medical Sciences, Chiang Mai University, Chiang Mai 50200, Thailand; tanawat_yo@cmu.ac.th (T.Y.); aatit.p@cmu.ac.th (A.P.); samatchai.c@cmu.ac.th (S.C.); amornthep.jan@cmu.ac.th (A.J.); 2 Department of Radiologic Technology, Faculty of Associated Medical Sciences, Chiang Mai University, Chiang Mai 50200, Thailand; sompong.sriburee@cmu.ac.th; 3 Department of Anatomy, Faculty of Medicine, Chiang Mai University, Chiang Mai 50200, Thailand; ranida.quiggins@cmu.ac.th; 4 Department of Physical Therapy, College of Medicine, National Cheng Kung University, Tainan 70101, Taiwan; connie@mail.ncku.edu.tw

## Error in Table

In the original publication [1], a typo was retained in Table 1 as published regarding the reporting of the side of low back pain in the asymptomatic group. With respect to the side of low back pain, asymptomatic participants did not report low back pain; therefore, this variable is not applicable and should not have been reported for this group. The corrected Table 1 appears below. The authors state that the scientific conclusions are unaffected. This correction was approved by the Academic Editor. The original publication has also been updated.

## Figures and Tables

**Table 1:** Participant demographics and baseline clinical characteristics.

Characteristics	Asymptomatic (Mean ± SD)	NSLBP (Mean ± SD)
Age, years	32.00 ± 11.28	30.56 ± 8.06
Sex	M: 8 (50%), F: 8 (50%)	M: 8 (50%), F: 8 (50%)
BMI	22.19 ± 2.87	21.84 ± 1.64
Leg dominance (right side n, %)	12 (75%)	11 (68.75%)
Side of low back pain (n, %)		
Unilateral	-	10 (62.5%)
Bilateral	-	6 (37.5%)
ODI Score	-	30.77 ± 7.35
Low back pain intensity (VAS Scale)	-	6.52 ± 0.84

M = male, F = female, BMI = body mass index, ODI = Oswestry Disability Index, VAS = Visual Analog Scale, Asymptomatic = asymptomatic control group, NSLBP = non-specific low back pain group.
